# Incidence of new-onset hypertension before, during, and after the COVID-19 pandemic: a 7-year longitudinal cohort study in a large population

**DOI:** 10.1186/s12916-024-03328-9

**Published:** 2024-03-19

**Authors:** Valentina Trimarco, Raffaele Izzo, Daniela Pacella, Ugo Trama, Maria Virginia Manzi, Angela Lombardi, Roberto Piccinocchi, Paola Gallo, Giovanni Esposito, Gaetano Piccinocchi, Maria Lembo, Carmine Morisco, Francesco Rozza, Gaetano Santulli, Bruno Trimarco

**Affiliations:** 1 Department of Neuroscience, Reproductive Sciences, and Dentistry, “Federico II” University, Naples, Italy; 2 Department of Advanced Biomedical Sciences, “Federico II” University, Naples, Italy; 3 Department of Public Health, “Federico II” University, Naples, Italy; 4 Pharmaceutical Department of Campania Region, Naples, Italy; 5 Department of Microbiology and Immunology, Fleischer Institute for Diabetes and Metabolism (FIDAM), Albert Einstein College of Medicine, New York City, NY USA; 6 “Luigi Vanvitelli” Hospital, Naples, Italy; 7 COMEGEN Primary Care Physicians Cooperative, Italian Society of General Medicine (SIMG), Naples, Italy; 8 International Translational Research and Medical Education (ITME) Consortium, Academic Research Unit, Naples, Italy; 9 Italian Society for Cardiovascular Prevention (SIPREC), Rome, Italy; 10 Department of Medicine, Wilf Family Cardiovascular Research Institute, Einstein-Mount Sinai Diabetes Research Center (ES-DRC), Albert Einstein College of Medicine, 1300 Morris Park Avenue, New York City, NY 10461 USA

**Keywords:** Blood pressure, Cardiovascular medicine, COVID-19, Epidemiology, Hypertension, Public health, SARS-CoV-2

## Abstract

**Background:**

While the augmented incidence of diabetes after COVID-19 has been widely confirmed, controversial results are available on the risk of developing hypertension during the COVID-19 pandemic.

**Methods:**

We designed a longitudinal cohort study to analyze a closed cohort followed up over a 7-year period, i.e., 3 years before and 3 years during the COVID-19 pandemic, and during 2023, when the pandemic was declared to be over. We analyzed medical records of more than 200,000 adults obtained from a cooperative of primary physicians from January 1, 2017, to December 31, 2023. The main outcome was the new diagnosis of hypertension.

**Results:**

We evaluated 202,163 individuals in the pre-pandemic years and 190,743 in the pandemic years, totaling 206,857 when including 2023 data. The incidence rate of new hypertension was 2.11 (95% C.I. 2.08–2.15) per 100 person-years in the years 2017–2019, increasing to 5.20 (95% C.I. 5.14–5.26) in the period 2020–2022 (RR = 2.46), and to 6.76 (95% C.I. 6.64–6.88) in 2023. The marked difference in trends between the first and the two successive observation periods was substantiated by the fitted regression lines of two Poisson models conducted on the monthly log-incidence of hypertension.

**Conclusions:**

We detected a significant increase in new-onset hypertension during the COVID-19 pandemic, which at the end of the observation period affected ~ 20% of the studied cohort, a percentage higher than the diagnosis of COVID-19 infection within the same time frame. This observation suggests that increased attention to hypertension screening should not be limited to individuals who are aware of having contracted the infection but should be extended to the entire population.

**Supplementary Information:**

The online version contains supplementary material available at 10.1186/s12916-024-03328-9.

## Background

After the initial observation by Xie and colleagues [1] that COVID-19 is associated with an increase in cardiovascular disorders, numerous clinical studies and meta-analyses have confirmed an augmented incidence of acute coronary syndromes, myocarditis, pericarditis, heart failure, and arrhythmias [2–8]. Equally important, an increase in the onset of new diabetes and hypertension has been documented [9]. While the augmented incidence of new-onset diabetes after COVID-19 has been thoroughly investigated and confirmed by numerous studies [10–15], less information is available regarding the risk of developing hypertension during the COVID-19 pandemic.

A retrospective study conducted by Tim Duong and collaborators [16] on patients with COVID-19 and patients with influenza has recently shown that after 6 months of observation, the incidence of new hypertension was detected in 20.6% of COVID-19 patients who had been hospitalized and in 10.85% of COVID-19 patients who did not require hospitalization [16]. The authors concluded that the risk of developing hypertension in these patients is more than double compared to patients hospitalized for influenza and 50% higher than the risk of patients with influenza who were not hospitalized [16]. In light of these results, the authors suggested the need for careful screening of patients who have contracted COVID-19 to limit both the health consequences for patients and healthcare costs.

However, these findings seem to be in contrast with another study that aimed at defining the risk of developing cardiovascular, neurological, psychiatric, or autoimmune diseases in 26,499 adults who survived COVID-19 hospitalization [17]. This study has shown that after a longer follow-up (1 year), the only condition that presents an increased risk, compared to patients hospitalized for influenza or for sepsis, is venous thromboembolism, while there are no differences for all the other considered pathologies, including hypertension [17]. Hence, the authors conclude that the so-called post-COVID-19 sequelae should be essentially attributed to the severity of the disease induced by the virus and not to the virus itself.

Since the above-mentioned studies had different follow-up periods (6 months vs. 12 months), a possible hypothesis to account for the discrepancy between the results of these two investigations could be that the increased risk of developing hypertension after COVID-19 diminishes over time, so that at the 1-year follow-up such risk is no longer different from the one induced by other types of infections. In order to clarify this issue and reconcile these apparently conflicting observations, we have analyzed the incidence of new hypertension in the general population, examining the phenomenon in the 3 years before COVID-19 (i.e., 2017–2019), in the 3 years in which the pandemic developed (i.e., 2020–2022), and in 2023, when the pandemic was declared to be over [18].

Notably, the data currently available in the literature merely refer to comparisons between groups of patients who certainly had a positive SARS-CoV-2 test vs. individuals with similar demographic characteristics who did not have COVID-19, thus assessing the effects of the long-term individual COVID-19 infection (post-acute sequelae) [19]. However, it should be considered that the attenuation of the severity of COVID-19 symptoms and the possibility of autonomously performing less-sensitive tests has resulted in some COVID-19 infections not being diagnosed [20, 21], making the evaluation of this complex phenomenon, and consequently its aftermaths on the public health organizations of different countries, less reliable.

In the present study, we specifically analyzed the impact of the pandemic on the incidence of new-onset hypertension in a population of individuals residing in the city of Naples in Southern Italy, followed by their primary care physicians from 2017 to 2023. We performed a cohort study assessing the incidence of new hypertension, based on the diagnosis of hypertension and the prescription of antihypertensive therapy, using an interrupted time series (ITS) approach to compare the 3-year period before the COVID-19 pandemic (i.e., 2017–2019) to a period of the same time length during the pandemic (i.e., 2020–2022), and to the year 2023.

To test the hypothesis that hypertension appearing after COVID-19 infection may be temporary and not persistent, we also evaluated the duration of prescriptions of antihypertensive medications in individuals with newly developed hypertension comparing the first and the second triennium. To rule out that the potential shorter duration in the second triennium (i.e., 2020–2022) was due to contingent reasons rather than the loss or disappearance of elevated blood pressure values, we also compared the duration of antihypertensive medication prescriptions during the two study periods for hypertensive individuals already present on January 1, 2017.

## Methods

### Study design

In this longitudinal cohort study, we harnessed data obtained from COMEGEN (*COoperativa di MEdicinaGENerale*: General Medicine Cooperative), an association of primary physicians in Naples (Local Health Authority of the Italian Ministry of Health, *ASL Napoli 1 Centro*) [22]. Founded in 1997, COMEGEN today includes 140 physicians who are all connected in an online network and implement the same computerized medical record software, creating a unique database containing medical records of more than 200,000 adult patients. These records are updated daily by these physicians, who upload all data concerning their outpatient activities. Of note, the territorial distribution of individuals assisted by these primary physicians is similar to the one of the city population recorded by the Italian National Institute of Statistics (ISTAT), with no differences in terms of geographic area or aggregation by age [22, 23]. The COMEGEN database collects diagnoses according to the International Classification of Diseases 10 (*ICD-X*) recorded by primary care providers, also using standardized codes for all prescribed diagnostic assessments. Pharmaceutical prescriptions are recorded with the date, commercial name, and active ingredients, alongside the quantities and methods of administration. Additionally, the database includes information on vital parameters, weight, height, BMI, waist circumference, chronic conditions, medical visits, hospitalizations, emergency department visits, prescription drug dispensations, testing and vaccinations (including for COVID-19), date, and cause of death. All these precious pieces of information allow for real-time provision of data related to the management of patients in terms of processes and outcomes, use of drugs, diagnostic investigations, and the complexity and comorbidities of the assisted population [23]. Moreover, an accurate evaluation of person-time is crucial for calculating incidence rates and the COMEGEN allows such assessment, by specifying when each individual has been entered in the database and started contributing data to the cohort, as well as the dates of death, end of follow-up, and end of observation.

We collected data from January 1, 2017, to December 31, 2023. The COMEGEN database provided demographic and clinical data. Laboratory measurements and medication data were available, as well. Information on COVID-19 was obtained from the COVID-19 shared data resource of the Campania Region. This cohort study followed the “Strengthening the Reporting of Observational Studies in Epidemiology” (STROBE) reporting guidelines. The Ethics Board at the “*ASL Napoli 1 Centro*” reviewed and approved this study and granted a waiver of informed consent.

The exposure was the beginning of the COVID-19 pandemic and the primary outcome was newly diagnosed hypertension. At the beginning of the 7-year observation period, we removed from the two study cohorts all individuals with a record of systolic blood pressure > 140 mmHg and/or diastolic blood pressure > 90 mmHg, or a previous diagnosis of hypertension as defined by the *ICD-10* (code I-10), or prescription record of anti-hypertensive medications for more than 30 days. We performed a single cohort study assessing the incidence of hypertension using an ITS approach with the same population followed for 3 years before and 3 years during the COVID-19 pandemic and 1 year when the pandemic was declared over. Only individuals whose information about hypertension history and in which demographic and clinical data were entirely available for the 7 years were included in the study. The diagnoses of other disorders, including hypercholesterolemia, hypertriglyceridemia, cancer, and chronic obstructive pulmonary disease, were made using ICD-10 codes*.*

The duration of prescriptions of antihypertensive medications in newly developed hypertension in the first and second triennium was computed considering the time elapsed between the diagnosis of hypertension and the last prescription of medications recorded. For both observation periods, when the last recorded prescription was obtained later than the end of the considered triennium, the individual was considered under medication for the whole 3-year period. To account for the fact that the prescription included 30 days of medication, individuals were considered as still under medication until 30 days after the last prescription was recorded.

### Statistical analysis

Continuous variables were summarized using mean (and standard deviation) and with median and interquartile range (IQR). Normality was evaluated with the Kolmogorov–Smirnov and with the visual inspection of density plots. New onset hypertension was reported as incidence per 100 person-years.

An ITS analysis on the period between January 2017 and December 2022 was performed to investigate potential changes in the trend of hypertension incidence in the pandemic years using an Autoregressive Integrated Moving Average (*ARIMA*) model. Our hypothesis was that exposure to COVID-19 over time could lead to a consistent increase in the incidence trend (ramp term) without significant short-term changes (step term). Stationarity was assessed using the augmented Dickey-Fuller test. Values of *p* (autoregressive order), *d* (degree of differencing), and *q* (moving average order) for the *ARIMA* model to reduce the impact of autocorrelation were selected comparing the models’ performances evaluated with the Akaike Information Criterion (AIC). To calculate the difference in the incidence trends between the pre-pandemic years (January 1, 2017, up to December 31, 2019) and the pandemic years (January 1, 2020, up to December 31, 2022) two separate log-linear models (Poisson regression models) were fitted, assuming a linear relationship between the log-incidence rate and time. The goodness-of-fit was evaluated by inspecting the residual plot and the qq plot to assess the normality of the residuals. In order to investigate whether the identified trend remained stable after the pandemic, we fitted the ITS model and the log-linear models on the period between January 2017 and December 2023, thus including data from 2023, when the pandemic was declared over.

The difference in the duration of the medical prescription between the group of individuals diagnosed with hypertension in 2017–2019 or in 2020–2022 was evaluated with the Wilcoxon rank sum test. For model estimates, 95% confidence intervals were also reported. Analyses were performed using the statistical software R, version 4.3.0. A two-tailed *p* < 0.05 was considered significant for all analyses.

### Role of the funding source

The funder of the study had no role in study design, data collection, data analysis, data interpretation, or writing of the report. R.I., D.P., and B.T. had access to the dataset; G.S. and B.T. had final responsibility for the decision to submit for publication. All authors vouch for the completeness and accuracy of the data.

## Results

**Fig. 1:**
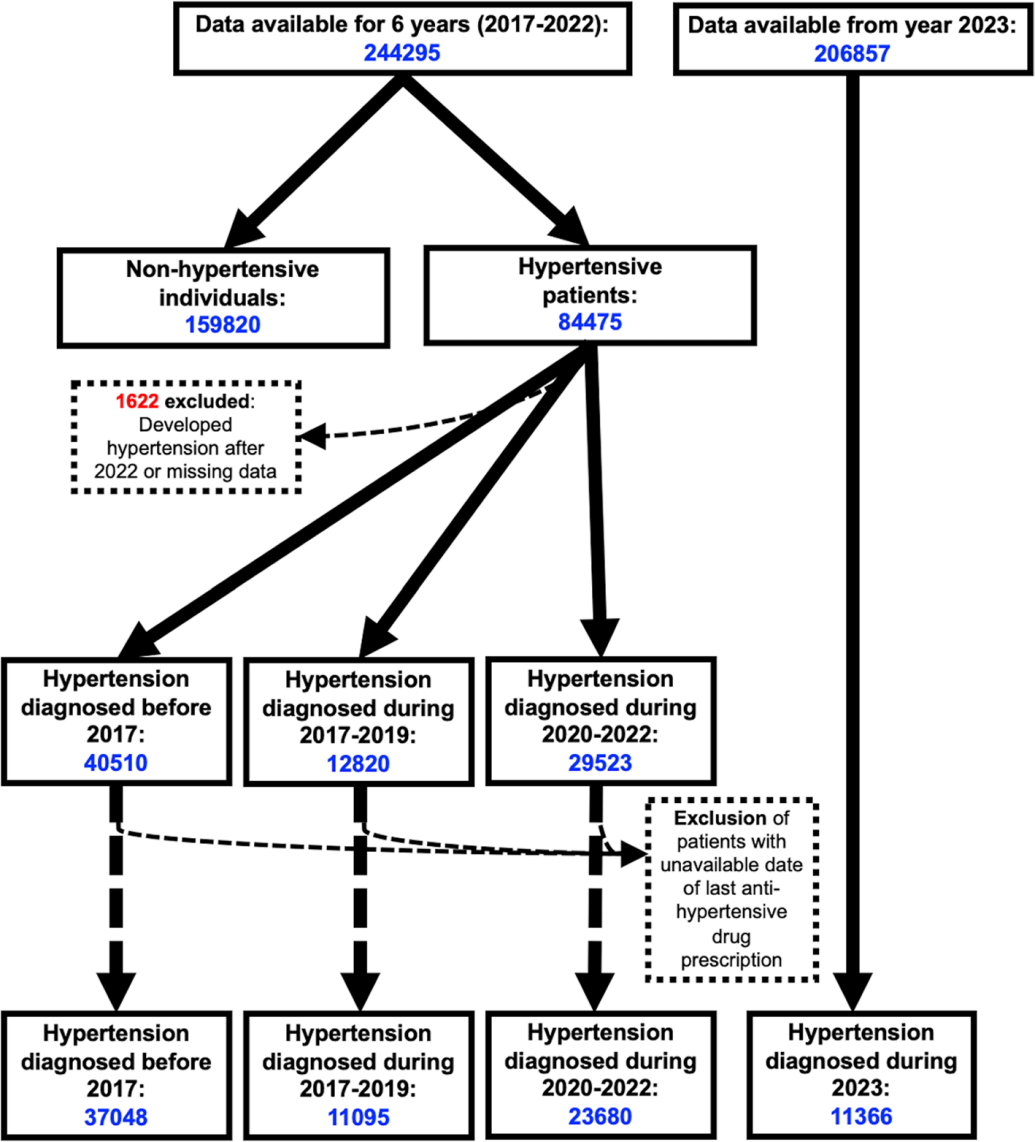
Flow chart of the study

From January 1, 2017, to December 31, 2022, a total of 244,295 individuals were observed (Fig. 1); 25,931 patients had a positive test for COVID-19 between April 1, 2020, and December 31, 2022. The main demographic and health characteristics of our study cohort are reported in Table 1.

**Table 1:** Demographic characteristics of the total cohort. Data presented as mean (SD) and confidence interval for continuous variables and as absolute frequency (percentage) for categorical variables

*Variable*	*N* = *202,163*	*95% CI*
Age (years)	47.7 (20)	47.7, 47.8
Glycemia (mg/dl)	91.7 (14)	91.5, 91.8
Hb1Ac (%)	6.04 (0.82)	6.03, 6.05
Sex		
F	108,062 (54%)	53%, 54%
M	93,530 (46%)	46%, 47%
Current or former smoking		
NO	41,705 (71%)	71%, 72%
YES	16,650 (29%)	28%, 29%
BMI (kg/m^2^)	27.2 (185.3)	26, 29
Creatinine (mg/dl)	0.853 (0.19)	0.851, 0.855
ALT (mU/ml)	19.9 (8)	19.9, 20
AST (mU/ml)	20.2 (6)	20.1, 20.2
Cancer		
NO	167,163 (82.7%)	82.5%, 82.9%
YES	35,000 (17.3%)	17.1%, 17.5%
COPD		
NO	197,551 (97.7%)	97.7%, 97.8%
YES	4612 (2.3%)	2.2%, 2.3%
Hypercholesterolemia		
NO	183,161 (90.6%)	90%, 91%
YES	19,002 (9.4%)	9.3%, 9.5%
Hypertriglyceridemia		
NO	200,266 (99.1%)	99.0%, 99.1%
YES	1897 (0.9%)	0.90%, 0.98%
COVID-19		
NO	176,232 (87.2%)	87.0%, 87.3%
YES	25,931 (12.8%)	12.7%, 13%

**Fig. 2:**
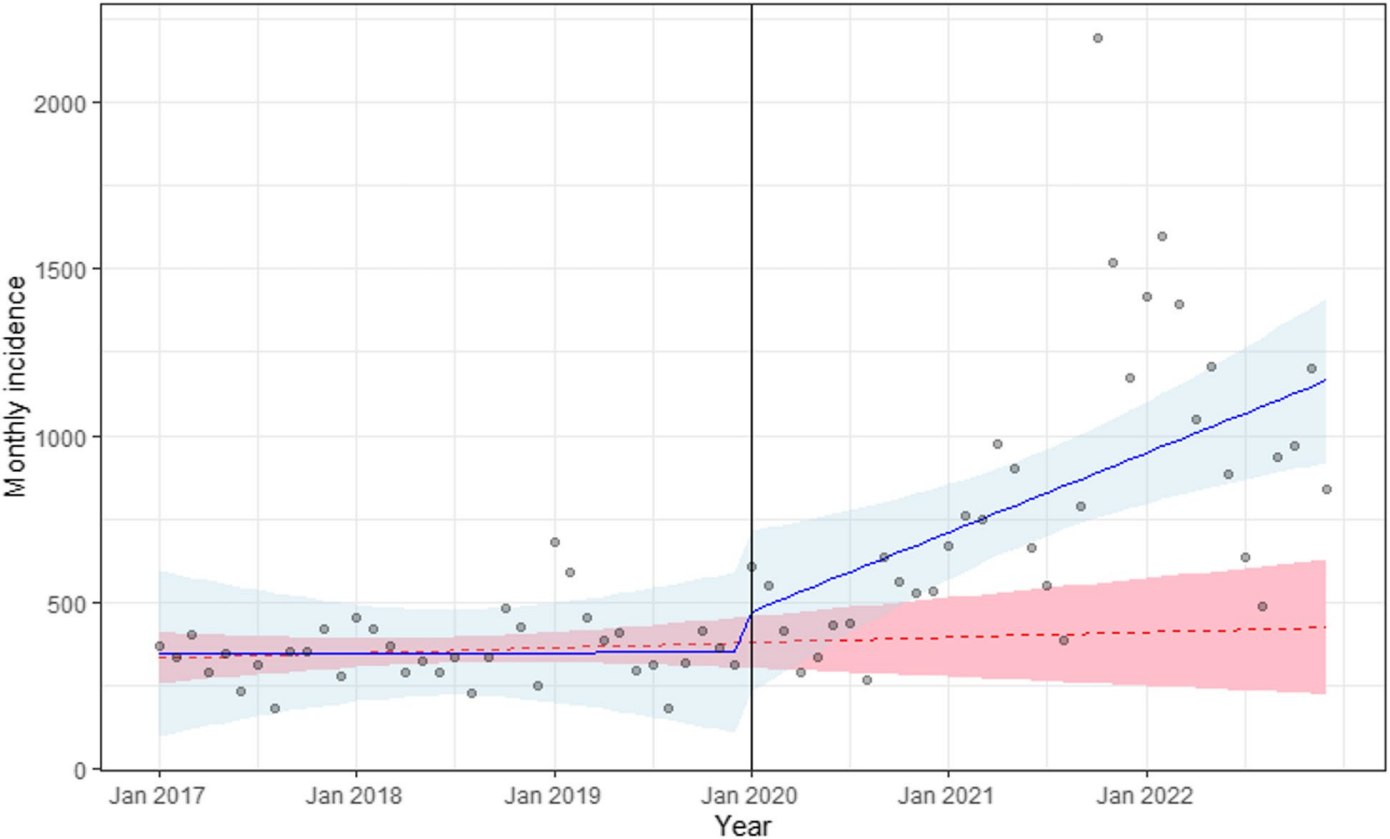
Interrupted time series (ITS) approach comparing the incidence of a new diagnosis of hypertension in the 3-year period before the COVID-19 pandemic (i.e., 2017–2019) to the 3 years during the COVID-19 pandemic (i.e., 2020–2022). The blue line represents the observed incidence with 95% confidence bands; the red dashed line represents the counterfactual line with 95% confidence bands

The absolute count of new diagnoses of hypertension in the whole observation period (2017–2022) was 42,343, with 12,820 new diagnoses in the period before the pandemic (2017–2019), 29,523 new diagnoses during the pandemic (2020–2022), and 11,366 new diagnoses in 2023 alone. The incidence rate of new hypertension was 2.11 (95% C.I. 2.08–2.15) per 100 person-years in the years 2017–2019, increasing to 5.20 (95% C.I. 5.14–5.26) in the period 2020–2022 (RR = 2.46) and to 6.76 (95% C.I. 6.64–6.88) in 2023. Table 2 reports the characteristics of individuals diagnosed with new hypertension in the period 2017–2019 and in the period 2020–2022, while the data reported in Table 3 and Additional file 1 (Figures S1–3) show the results of stratification analysis by age (< 65 or ≥ 65 years old), sex, and cancer. These latter analyses suggest that the increase in new-onset hypertension during the pandemic was detectable in the whole population but it was more marked in subjects aged > 65 years and in those without cancer. The ITS model conducted on the data revealed the best performance and minimized autocorrelation with ARIMA values *p* = 0, *d* = 0, *q* = 2. Figure 2 displays the regression line of the observed data against the counterfactual line. The coefficients estimated with the ITS model evidenced a significant effect of time during the pandemic on the incidence trend of hypertension, with a significant change in the slope during the three pandemic years (“ramp term” coefficient *b* = 19.71, *p* = 0.031). The effect of time before the pandemic was not significant (overall time effect coefficient *b* = 0.07, *p* = 0.990), indicating that the trend of the incidence of hypertension was stable before the beginning of the pandemic. While the effect of the pandemic over time was observed, there was no immediate change in the incidence of hypertension at the beginning of the pandemic period, as hypothesized (“step term” coefficient 103.01, *p* = 0.538).

**Table 2:** Demographic characteristics of individuals diagnosed with hypertension in the period 2017–2019 and in the period 2020–2022. Data presented as mean (SD) and confidence interval for continuous variables and as absolute frequency (percentage) for categorical variables

*Variable*	*2017–2019 N* = *12,820*	*95% CI*	*2020–2022 N* = *29,523*	*95% CI*
Age (years)	66 (14)	65.9, 66.4	66 (14)	66, 67
Glycemia (mg/dl)	97 (15)	96, 97	98 (15)	97, 98
Hb1Ac (%)	6.17 (0.77)	6.1, 6.2	6.23 (0.78)	6.2, 6.3
Sex				
F	6830 (53%)	52%, 54%	16,078 (54%)	54%, 55%
M	5978 (47%)	46%, 48%	13,435 (46%)	45%, 46%
Current or former smoking				
NO	4924 (73%)	71%, 74%	6496 (73%)	72%, 74%
YES	1867 (27%)	26%, 29%	2388 (27%)	26%, 28%
BMI (kg/m^2^)	26.6 (3.7)	26, 27	26.5 (3.5)	26, 27
Creatinine (mg/dl)	0.89 (0.20)	0.89, 0.90	0.90 (0.21)	0.90, 0.91
ALT (mU/ml)	20 (8)	20, 21	20 (8)	20.0, 20.4
AST (mU/ml)	21 (6)	20, 21	20 (6)	20, 21
Cancer				
NO	8987 (70%)	69%, 71%	23,470 (79%)	79%, 80%
YES	3833 (30%)	29%, 31%	6053 (21%)	20%, 21%
COPD				
NO	12,072 (94.2%)	94%, 95%	27,504 (93.2%)	92.9%, 93.4%
YES	748 (5.8%)	5.4%, 6.3%	2019 (6.8%)	6.6%, 7.1%
Hypercholesterolemia				
NO	9812 (77%)	76%, 77%	23,224 (79%)	78%, 79%
YES	3008 (23%)	23%, 24%	6299 (21%)	21%, 22%
Hypertriglyceridemia				
NO	12,433 (97%)	96.7%, 97.3%	28,976 (98.1%)	98.0%, 98.3%
YES	387 (3%)	2.7%, 3.3%	547 (1.9%)	1.7%, 2.0%
COVID-19				
NO			26,019 (88%)	87.8%, 88.5%
YES			3504 (12%)	11.5%, 12.2%

**Table 3:** Incidence of new-onset hypertension per 100 person-years by sex, age group, and cancer

	***Incidence per 100 person-years in 2017–2019 (95% C.I.)***	***Incidence per 100 person-years in 2020–2022 (95% C.I.)***	***Relative risk 2020–2022 vs 2017–2019 (95% C.I.)***	***p-value***
Male	2.16 (2.10, 2.21)	4.56 (4.48, 4.63)	2.11 (2.05, 2.18)	< 0.001
Female	2.13 (2.08, 2.18)	5.31 (5.23, 5.39)	2.49 (2.42, 2.56)	< 0.001
Age < 65	1.26 (1.23, 1.29)	2.81 (2.77, 2.86)	2.23 (2.17, 2.30)	< 0.001
Age ≥ 65	5.43 (5.30, 5.55)	15.50 (15.28, 15.71)	2.85 (2.78, 2.93)	< 0.001
Cancer: yes	3.70 (3.59, 3.82)	6.50 (6.34, 6.66)	1.76 (1.69, 1.82)	< 0.001
Cancer: no	1.81 (1.77, 1.85)	4.96 (4.90, 5.02)	2.74 (2.67, 2.80)	< 0.001

**Fig. 3:**
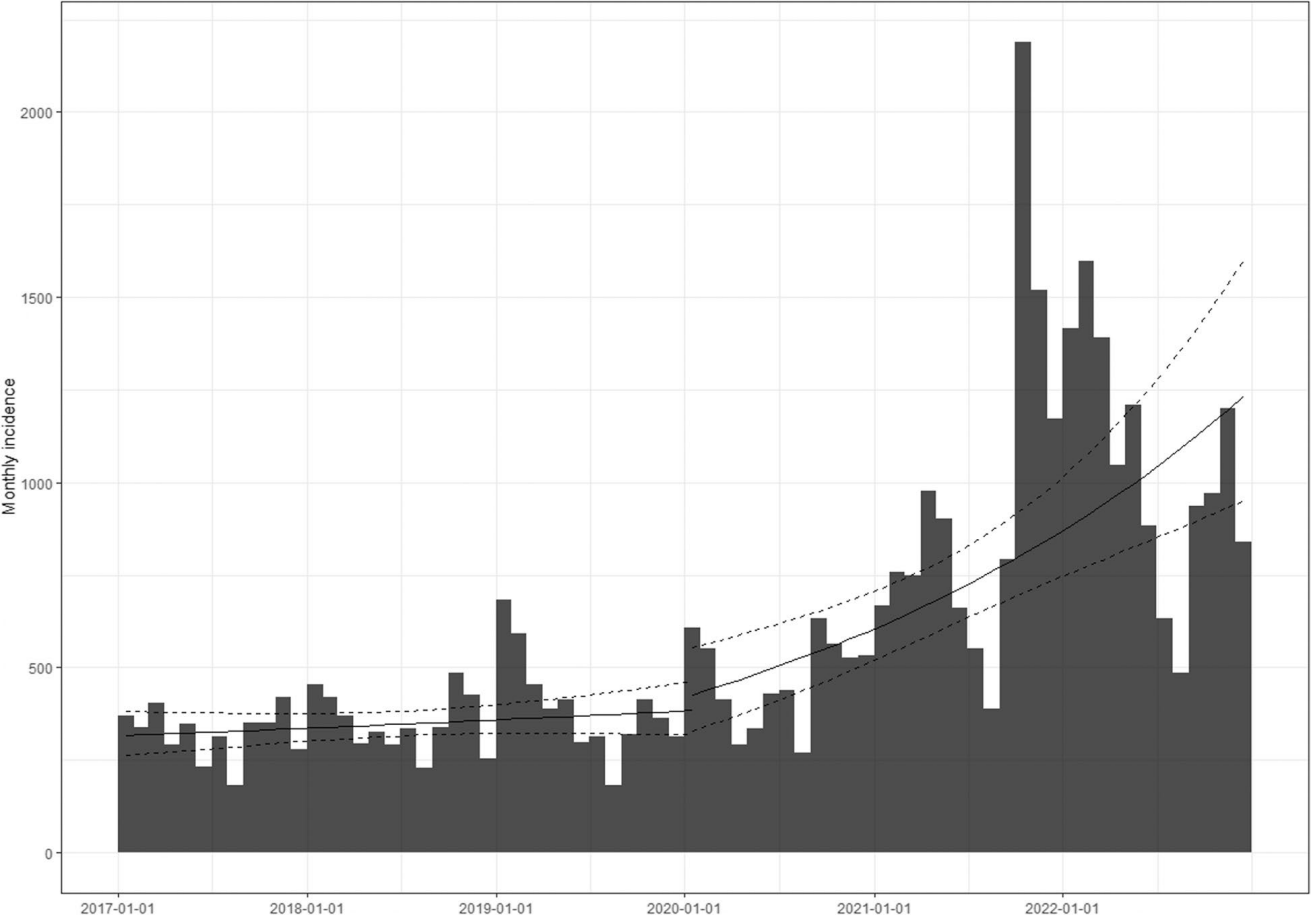
Monthly incidence of hypertension in the complete cohort during the 6-year observation period (2017–2022) and linear regression lines estimated with the two log-linear models fitted on each time period. Dashed lines represent the 95% confidence interval

The marked difference in trends between the two observation periods is substantiated by the fitted regression lines of the two Poisson models conducted on the monthly log-incidence of hypertension (Fig. 3). In the pre-pandemic period, in line with the results of the ITS analysis, the slope of the trend of the estimated monthly incidence rate was not significant (0.005, 95% C.I. − 0.003, 0.014) whereas in the pandemic period it was significantly positive (0.030; 95% C.I. 0.017, 0.042).

**Fig. 4:**
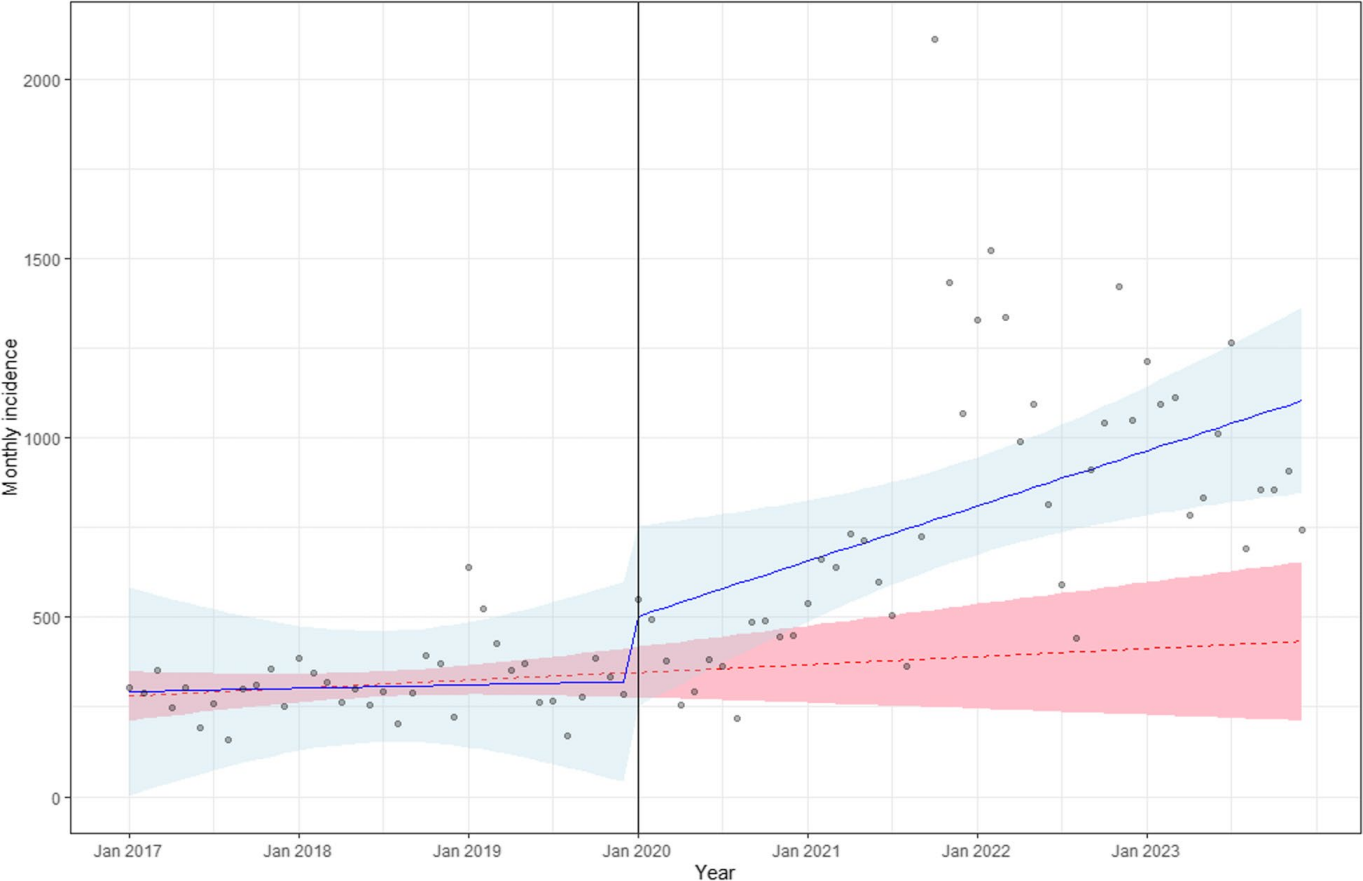
Interrupted time series (ITS) approach comparing the incidence of a new diagnosis of hypertension in the 3-year period before the COVID-19 pandemic (i.e., 2017–2019) to the 3 years during the COVID-19 pandemic (i.e., 2020–2022) and including the year 2023, when the pandemic was declared over. The blue line represents the observed incidence with 95% confidence bands; the red dashed line represents the counterfactual line with 95% confidence bands

**Fig. 5:**
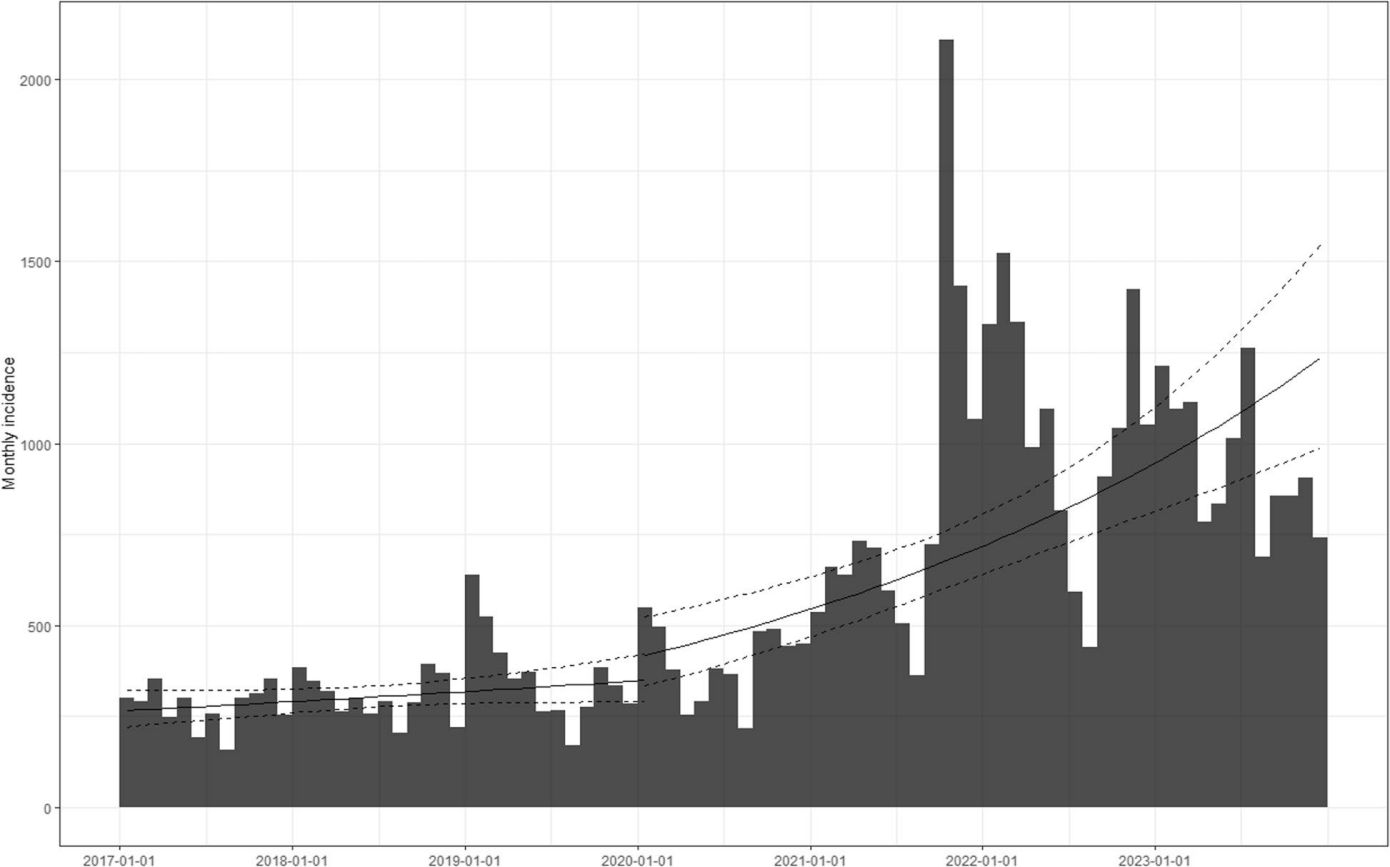
Monthly incidence of hypertension in the complete cohort during the 7-year observation period (2017–2023) and linear regression lines estimated with the two log-linear models fitted on each time period. Dashed lines represent the 95% confidence interval

The additional confirmatory ITS model conducted on the period 2017–2023 displayed in Fig. 4 shows consistent results and again a significant “ramp term” coefficient (ARIMA values *p* = 0, *d* = 0, *q* = 1, “ramp term” coefficient* b* = 12.60, *p* = 0.048, overall time effect coefficient* b* = 1.41, *p* = 0.785, “step term” coefficient 128.16, *p* = 0.355), while the log-linear model conducted on the period 2020–2023 shows a slope with a still significantly increasing trend (0.027, 95% C.I. 0.018–0.037) (Fig. 5).

**Fig. 6:**
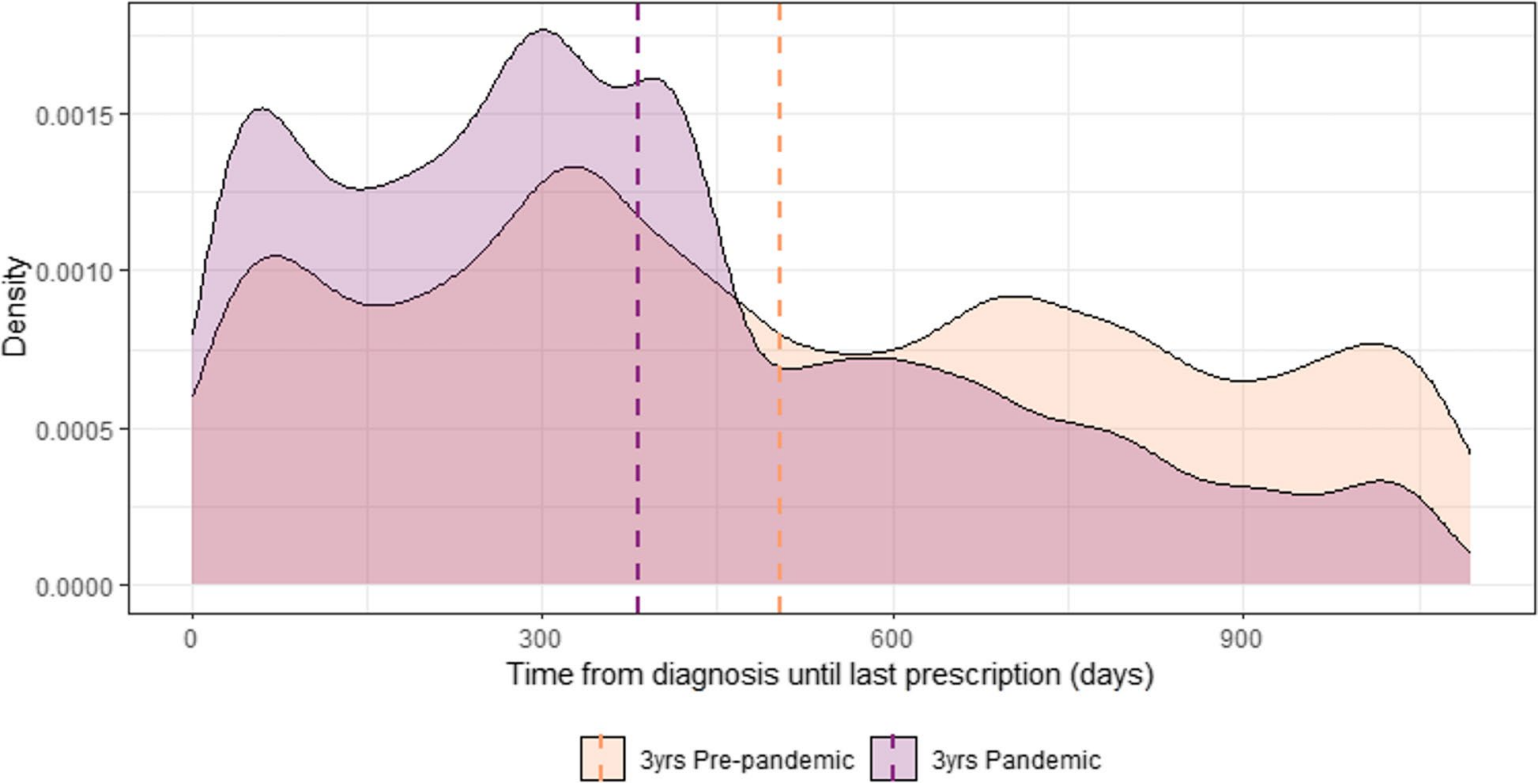
Density plots showing the frequency density of the absolute number of days covered by prescription in the subgroup of individuals diagnosed with hypertension in the 3 years before the pandemic (2017–2019: orange curve) and in the subgroup of individuals diagnosed in the 3 years during the pandemic (2020–2022: purple curve). Dashed lines represent the means for each group

**Fig. 7:**
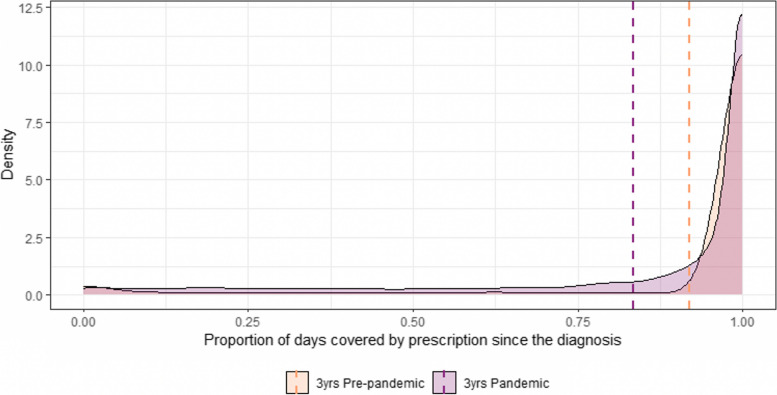
Density plots showing the frequency density of the proportion of days covered by prescription (measured as the absolute number of days under prescription over the total number of days from the diagnosis until the end of the observation period) in the subgroup of individuals diagnosed with hypertension in the 3 years before the pandemic (2017–2019: orange curve) and in the subgroup of individuals diagnosed in the 3 years during the pandemic (2020–2022: purple curve). Dashed lines represent the means for each group

The distributions of the days covered by medical prescription in the observation period for the individuals diagnosed with hypertension during the pre-pandemic years and during the pandemic years are depicted in Fig. 6. To compare the two distributions, we have computed the proportion between the number of days covered by anti-hypertensive medical prescription over the total time elapsed between the diagnosis of hypertension and the end of the 3-year observation period. To account for the fact that the prescription included 30 days of medication, individuals were considered as still under medication until 30 days after the last prescription was recorded. The proportion of days under medical prescription (measured as the absolute number of days under prescription over the total number of days from the diagnosis until the end of the observation period) in the group diagnosed before the pandemic was significantly higher (*p* < 0.001) than in the group diagnosed in the years during the pandemic as shown in Fig. 7 (3-year pre-pandemic period mean 0.92, S.D. 0.24; median 1.00, IQR 0.00 vs. 3-year pandemic period mean 0.83, S.D. 0.28; median 0.99; IQR 0.20). The number of patients with 100% prescription coverage in the group diagnosed in the pre-pandemic triennium was 9656 out of 11,095 (87%) while only 10,726 out of 23,680 (45%) of those diagnosed during the pandemic years were covered for the whole period after diagnosis.

Furthermore, we measured the proportion of days under medical prescription in the two periods for the 37,048 patients with a diagnosis of hypertension obtained before January 1, 2017. Even in this cohort, as shown in Fig. 7, the proportion of days under coverage was significantly higher (*p* < 0.001) in the 3 years before the pandemic (mean 0.95, S.D. 0.15; median 1.00, IQR 0.00) than in the 3 years during the COVID-19 pandemic (mean 0.77, S.D. 0.36, median 0.99, IQR 0.34). The number of patients with 100% prescription coverage in the pre-pandemic years was 32,507 (88%) while it decreased to 14,841 (40%) in the pandemic years.

## Discussion

To the best of our knowledge, the present study is the first to be performed on a large sample of the general population and not on databases of hospitals or outpatient clinics. Indeed, in Italy, all citizens have a primary care physician (family doctor) who follows them even if they do not have specific pathologies. Therefore, the database that we used includes not only patients with known illnesses but also individuals who do not have any disease (or at least are not aware of it). This situation currently applies to COVID-19 infection since the attenuation of symptoms and the lack of meticulous tracking of the infection have resulted in many individuals contracting the disease without being aware of it, or not reporting this condition to the health authorities. Indeed, observational retrospective studies cannot fully clarify the pathophysiological mechanisms of diseases but can define the social and economic burden they impose on the population. From this perspective, studies involving large populations are preferred over those conducted on hospitalized or outpatient individuals, whose results must be then extrapolated to the entire population.

Our results clearly demonstrate that COVID-19 pandemic is accompanied by a significant increase in the incidence of new permanent hypertension, with data even more alarming than those published by Duong and collaborators [16]: while these authors reported a maximum peak of 20.6% in patients hospitalized for COVID-19 and 10.85% in outpatients, which then had stabilized to ~ 10%, in our study the incidence of new hypertension continued to increase over the course of the 3 years of the pandemic. At the end of the observation period, it affected approximately 15% of our population, above all in subjects 65 years old and in those without a cancer diagnosis, a finding that was still evident in 2023. This observation is pivotal from an epidemiological perspective because it implies that increased attention to hypertension screening should be extended to the entire population instead of being limited to those who are aware of having contracted the SARS-CoV-2 infection.

The fact that we actually detected permanent hypertension is documented not only by the incidence of the diagnosis of hypertension according to the *ICD-X* I-10 code but also by the assessment of antihypertensive medication prescriptions. In fact, the decline in the percentage of therapeutic coverage in patients with a new diagnosis of hypertension during the pandemic compared to those in the pre-pandemic period is very similar to what is observed during the two study periods in patients with hypertension diagnosed before January 1, 2017, indicating that this phenomenon is due to environmental contingencies during the pandemic period rather than to the attenuation or disappearance of elevated blood pressure. Lastly, the observation that the increased incidence of new-onset hypertension was still detectable in 2023 is not surprising since the virus circulation was still high, so a new vaccination campaign was undertaken [18].

A major strength of our study is given by having access to data on the general population in the years before the pandemic and during the pandemic. Thus, we used an ITS approach on a single cohort with the only variation being the appearance of the pandemic in the second 3-year of follow-up (2020–2022) compared to the first period of observation (2017–1019) to analyze the impact of the pandemic in a broader force on the risk of developing arterial hypertension. When performing the “before *vs.* after” comparison, we also considered that the difference in the incidence of new-onset hypertension between the two periods can be multifactorial. First, we took into account the time effect, as an increased trend of hypertension incidence over time could have been expected even before the COVID-19 pandemic, especially with a relatively long follow-up period. However, the stable incidence during the first 3 years of observation and the sharp increase in the monthly incidence of new hypertension during the second half of the follow-up period makes this hypothesis unlikely. Secondly, with this approach, we also managed to overcome potential issues of lack of awareness of the disease, which has been a crucial limitation in previous studies due to the impossibility of ensuring that the control group was truly infection-free [24–27]. Moreover, the long study period may allow the possibility to generalize our findings to the immunity and viral characteristics of the current population, as it includes the evolution of viral strains, widespread SARS-CoV-2 reinfections, or administration of more than 2 doses of COVID-19 vaccines. Nevertheless, the observation of an increase in incident new persistent hypertension in the Campania Region, where the percent of people who were partially or fully vaccinated against COVID-19 has been very high [28], makes our findings particularly relevant by suggesting that the increased cardiovascular risk associated with COVID-19 might be extended for years and not limited to the acute phase of the infection.

Our study is not exempt from limitations. First, we cannot rule out that the larger increase in the incidence of new persistent hypertension recorded in our study during the COVID-19 pandemic may also include the effects of influenza infection on this parameter [13, 14]. However, this possibility does not modify the relevance of our conclusions since, by encompassing the entire years of follow-up, we can assume that the role of influenza in determining hypertension should be at least constant in the two periods, if not lower in the second triennium due to the use of masks and other measures implemented during the pandemic, which limited the incidence of influenza infection [29, 30]. Second, we acknowledge that our results do not allow any speculation on the pathophysiologic mechanisms underlying the increased rate of incident hypertension in the pandemic period, which could include direct effects of SARS-CoV-2 infection on endothelial cells, and indirect effects like stress, changes in diet and exercise and in cardiovascular prevention strategies, and access to health system during pandemic; other possibilities include persistent activation of RAAS and endothelial injury which have been reported among patients with COVID-19 and both are associated with blood pressure elevation [31–34]. Besides, consequences of severe COVID-19 including systemic hypoxia, acute respiratory distress, hyper-coagulation, sepsis, inflammation, metabolic stress, and cytokine storm may stress the cardiovascular system, eventually leading to blood pressure dysregulation [35–38]. Finally, the pandemic itself, but not necessarily the infection, might raise blood pressure via increased stress as a consequence of dramatic changes in health strategies, lifestyle, and economic status [39–47]. The pandemic crisis has caused great unrest in society and unprecedented changes in lifestyle, work, and social interactions [48]. The implementation of policies such as social distancing and the closure of gathering and interaction centers such as parks, cafes, schools, universities, etc., has had certain social consequences [49]. The adoption of smart work modalities by many industries and institutions may have exerted deleterious effect on health outcomes according to the results of a recently published paper which has demonstrated that after adjusting for sex, age, education, smoking, drinking, and body mass index, individuals who mostly sat at work had a 16% higher all-cause mortality risk, and a 34% increased mortality risk from cardiovascular disease compared with those who were mostly non-sitting at work [50], although the prevalence of arterial hypertension remained unchanged.

However, defining the exact mechanisms is beyond the scope of this epidemiologic study. Third, we trust that the positivity reported in our sample is greatly underestimated compared to the prevalence measured in the Campania Region, where Naples is located, since there have been more than 2.4 million cases of COVID-19 in the 3 years of the pandemic, and the total population of the Campania Region is between approximately 6 million. Consequently, since the proportion of individuals with a history of COVID-19 positivity is largely underreported in our dataset, we decided not to include this information in the model as this strategy would have introduced bias.

## Conclusions

We observed a significantly augmented incidence rate of new hypertension in the period 2020–2022 compared to the triennium 2017–2019, increasing from 2.11 to 5.2% (RR = 2.46). The difference in trends between the two periods was corroborated by the fitted regression lines of two Poisson models conducted on the monthly log-incidence of hypertension. In the pre-COVID-19 period, fully in line with the results of the ITS analysis, the slope of the trend of the estimated monthly incidence rate was not significant, whereas during the pandemic it was significantly positive. It remained positive in 2023, when the pandemic was declared over but virus circulation was still high [18]. The observation of an increased incidence of new-onset hypertension in the whole population during the pandemic suggests that hypertension screening should not be limited to individuals who are aware of having contracted the infection but should be extended to the entire population.

### Supplementary Information

**Additional file 1: Figure S1. **Monthly incidence of hypertension during the 6-year observation period (2017-2022) grouped by sex. **Figure S2. **Monthly incidence of hypertension during the 6-year observation period (2017-2022) by age group. **Figure S3. **Monthly incidence of hypertension during the 6-year observation period (2017-2022) by cancer prevalence.

## Data Availability

Data and material will be available upon reasonable request to the first authors.

## Abbreviations

BMI: Body mass index
COMEGEN: General Medicine Cooperative (*COoperativa di MEdicinaGENerale*)
COVID-19: Coronavirus disease 2019
ITS: Interrupted time series
SARS-CoV-2: Severe acute respiratory syndrome coronavirus 2
