# Supplementary material for: Incidence of new-onset hypertension before, during, and after the COVID-19 pandemic: a 7-year longitudinal cohort study in a large population
Source: BMC Med. 2024 Mar 19;22:127. doi: 10.1186/s12916-024-03328-9 (PMC10949764; doi:10.1186/s12916-024-03328-9)
Supplement: Supplementary file 1 — Additional file 1: Figure S1. Monthly incidence of hypertension during the 6-year observation period (2017-2022) grouped by sex. Figure S2. Monthly incidence of hypertension during the 6-year observation period (2017-2022) by age group. Figure S3. Monthly incidence of hypertension during the 6-year observation period (2017-2022) by cancer prevalence. [file 12916_2024_3328_MOESM1_ESM.pdf]

Additional File

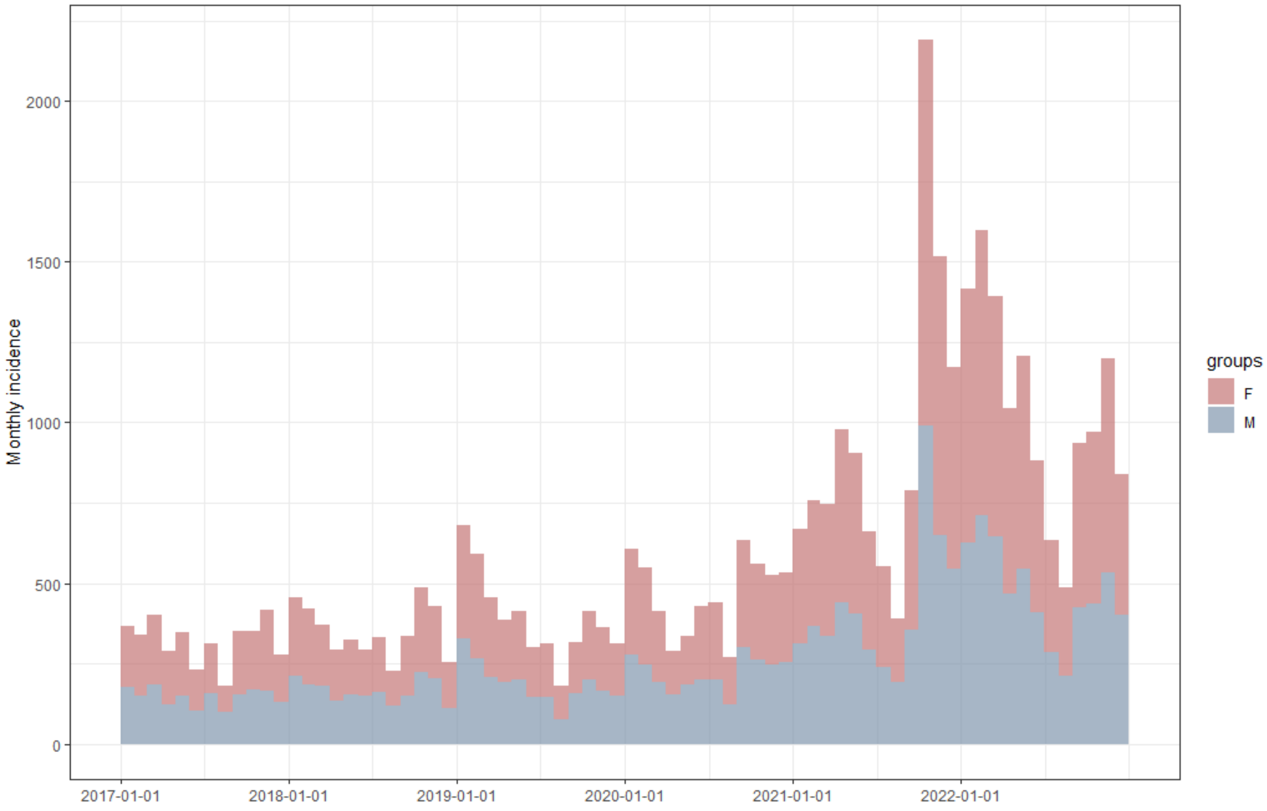

Figure S1

Monthly incidence of hypertension during the 6-year observation period (2017-2022) grouped by sex.

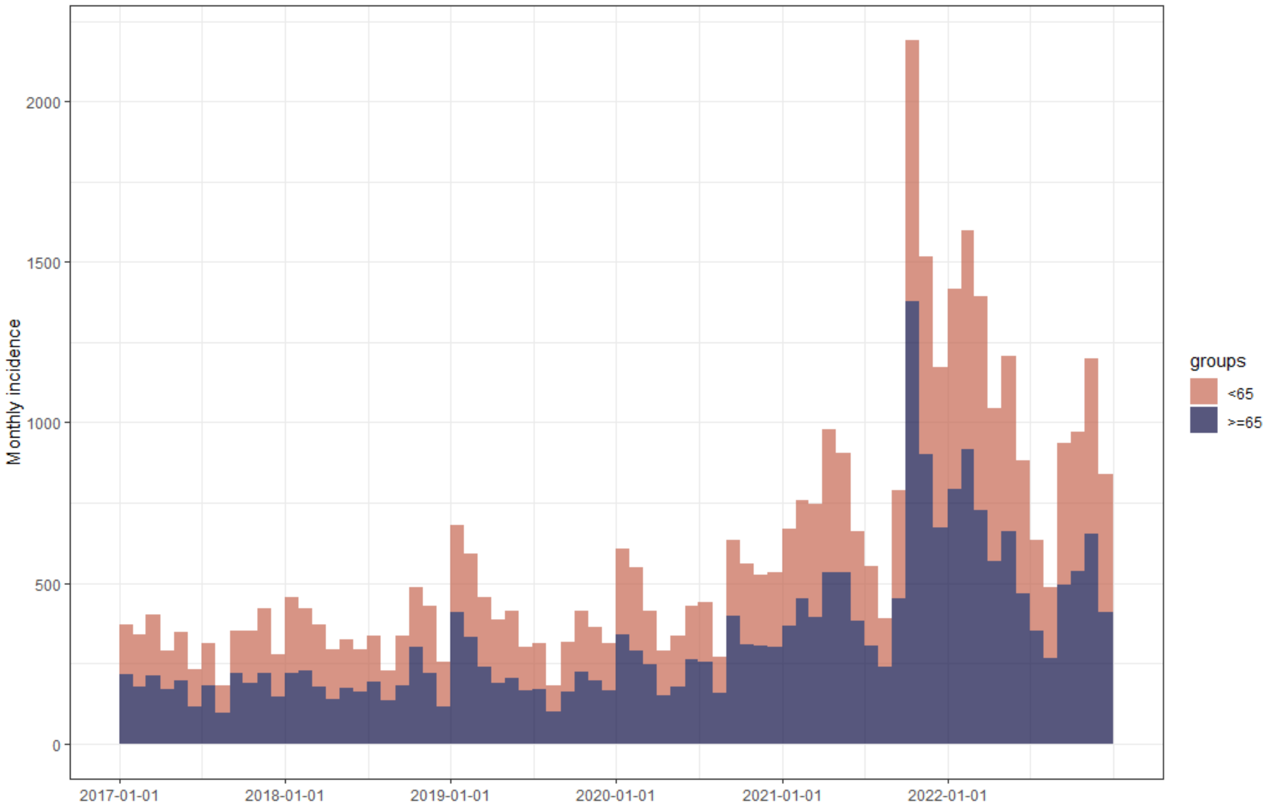

Figure S2

Monthly incidence of hypertension during the 6-year observation period (2017-2022) by age group.

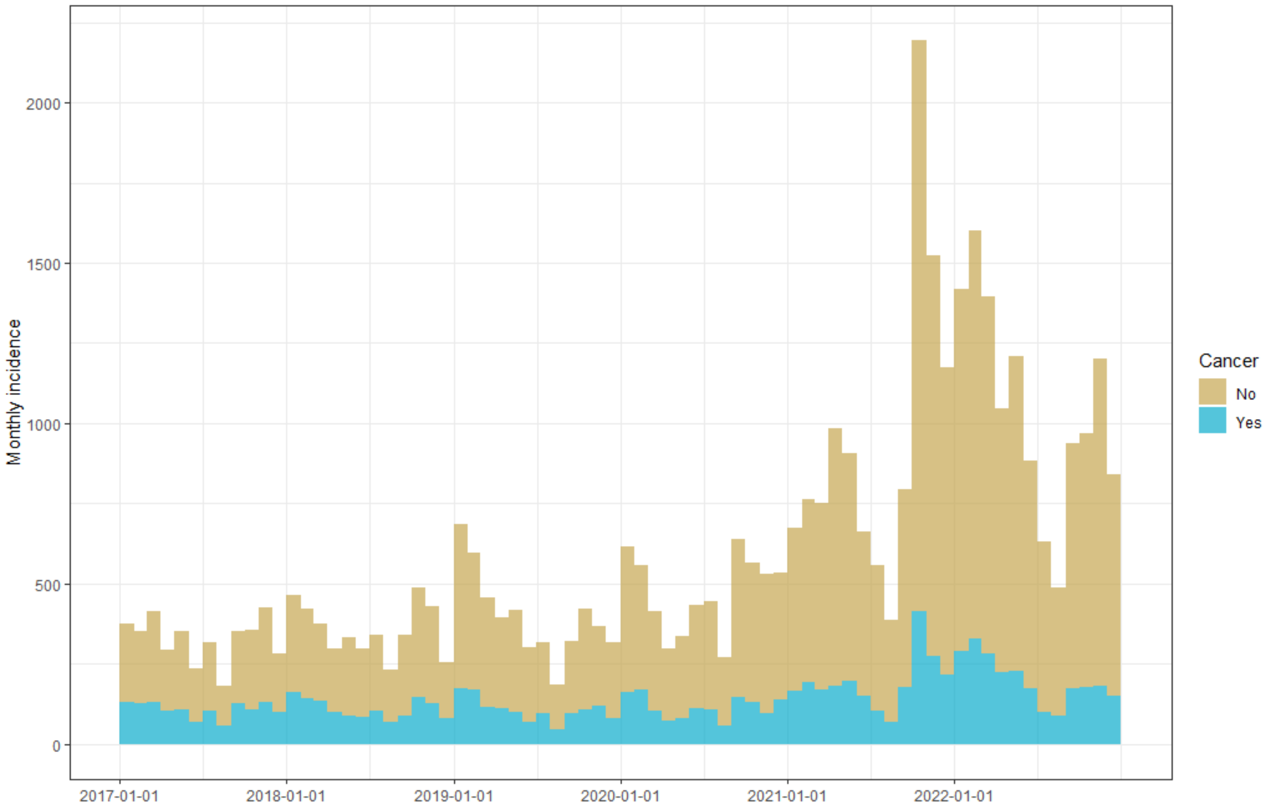

Figure S3

Monthly incidence of hypertension during the 6-year observation period (2017-2022) by cancer prevalence.
